# Thermotolerant yeasts selected by adaptive evolution express heat stress response at 30 °C

**DOI:** 10.1038/srep27003

**Published:** 2016-05-27

**Authors:** Luis Caspeta, Yun Chen, Jens Nielsen

**Affiliations:** 1 Centro de Investigación en Biotecnología, Universidad Autónoma del Estado de Morelos, MX-62209, Cuernavaca, Morelos, México; 2 Novo Nordisk Foundation Center for Biosustainability, Chalmers University of Technology, SE-41296 Gothenburg, Sweden; 3 Department of Biology and Biological Engineering, Chalmers University of Technology, SE-41296 Gothenburg, Sweden; 4 Novo Nordisk Foundation Center for Biosustainability, DK-2970 Hørsholm, Denmark; 5 Science for Life Laboratory, KTH Royal Institute of Technology, SE17165 Solna, Sweden

## Abstract

Exposure to long-term environmental changes across >100s of generations results in adapted phenotypes, but little is known about how metabolic and transcriptional responses are optimized in these processes. Here, we show that thermotolerant yeast strains selected by adaptive laboratory evolution to grow at increased temperature, activated a constitutive heat stress response when grown at the optimal ancestral temperature, and that this is associated with a reduced growth rate. This preventive response was perfected by additional transcriptional changes activated when the cultivation temperature is increased. Remarkably, the sum of global transcriptional changes activated in the thermotolerant strains when transferred from the optimal to the high temperature, corresponded, in magnitude and direction, to the global changes observed in the ancestral strain exposed to the same transition. This demonstrates robustness of the yeast transcriptional program when exposed to heat, and that the thermotolerant strains streamlined their path to rapidly and optimally reach post-stress transcriptional and metabolic levels. Thus, long-term adaptation to heat improved yeasts ability to rapidly adapt to increased temperatures, but this also causes a trade-off in the growth rate at the optimal ancestral temperature.

Changes in signaling networks to adjust gene expression programs and metabolic fluxes according to environmental change is a concomitant homeostatic response of cells when getting exposed to agents which can damage their macromolecules and desynchronize their metabolism^1^,^2^,^3^. Increased temperature causes changes in gene expression program consisting of an immediate modification in the transcription of “early stress response genes”, often referred to as the environmental stress response (ESR) which is a common response to many stresses^1^,^4^,^5^, followed by changes in the expression of genes specifically associated with the heat stress response^1^,^5^. According to the magnitude and prevalence of heat stress, these changes can be transient and finally adjusted to ensure that the cells can cope with the new temperature^5^. Sometimes, if the temperature change is mild, transcriptional programs are reset to the pre-stimulus value. As a result of the metabolic expenditure required for reestablishing homeostasis, many changes in metabolic fluxes simultaneously occur due to variations in energy charge and reduction/oxidation potentials, concomitant with a decrease in growth rate^1^,^6^,^7^,^8^,^9^.

The yeast *Saccharomyces cerevisiae* transiently changes the expression of thousand genes in response to a temperature increase. This response depends on the extent of temperature increase and on the proximity of the temperature to that being completely growth-inhibitory, i.e. ~40 °C^5^,^10^. Genes that encode proteins associated with rescuing unfolded proteins from degradation, restoring unfolded proteins, trehalose synthesis, recovering microfluidic-state of membranes, restoring DNA structure, protecting splicing from disruption, and protection against excessive energy consumption are usually overexpressed^10^,^11^,^12^,^13^,^14^. Genes with reduced expression are those encoding components of the ribosome and protein synthesis, as well as those involved in cell cycle progression. The central metabolism is adjusted through the activity of the cAMP-PK pathway, which decreases simultaneously with transcriptional changes, favoring trehalose synthesis in detriment of glycolytic fluxes, protein synthesis, and, lastly, the replication rate^10^,^13^. Prevalent high temperatures also impair proton shuttles in the mitochondrial membrane and thus the yeast is obligated to increase glycerol synthesis to adjust proton balance^6^,^7^,^15^. Heat also disrupts energy generation through reduced respiratory metabolism^6^, even though the oxygen uptake rate is increased^7^. Depending on the extent of heat stress, yeast can resume growth at slower rates, remain in replication senescence or activate the apoptotic program^10^,^14^,^16^. Interestingly, yeast survival to killing temperatures (>45 °C) can be improved by the activation of the ESR triggered through mild stresses –e.g. low pH, high osmolarity (KCl 1 M), elevated ethanol concentrations (5–8% V/V), mild temperature (37 °C), etc.^1^,^14^,^17^. This suggests that such a preadaptive response reduces cellular efforts to coordinate the homeostatic response.

Earlier we found that seven thermotolerant *S. cerevisiae* strains (TTSs) isolated from independent adaptive laboratory evolution (ALE) experiments at high temperature (39.5 °C), acquired single nucleotide variations (SNVs) in *ATP2/3* and *ERG3* genes^18^. All the SNVs were deleterious to the function of their encoded proteins. Consequently, the TTSs and the point-mutation versions of the wild type strain (WTS) were unable to consume respiratory carbon sources – only one mutation in *ERG3* did not cause this phenotype. The point mutation in *ATP3* caused a decreased fitness in the WTS at 30 °C and 40 °C, whereas the WTS with the point mutations in *ERG3*, which caused significant changes in sterol metabolism, recovered around 86% of the observed thermotolerant phenotype in the TTSs.

Sterols are critical for the formation of lipid “rafts” and regulation of membrane dynamics to maintain microfluidic state and to perform essential biological processes including cellular sorting, cytoskeleton organization, asymmetric growth and signal transduction^19^,^20^. In fact, the physical state of membranes contribute to the control of the heat-shock response^21^. For example, the addition of a membrane fluidizer or the hydrogenation of plasma membrane triggered the overexpression of heat shock proteins in *Synechocystis sp*.^22^,^23^. In *S. cerevisiae*, changes in the membrane lipid composition affected the expression of the HSP70 family and the *HSP82* gene which is a negative regulator of the heat-shock transcription factor HSF1, modifying the threshold of the heat shock response^24^,^25^. Altogether, these observations suggested that the TTSs could display very different gene expression and metabolic programs compared to the WTS in cultivations at the ancestral and high temperatures, allowing the TTSs to robustly control heat stress. To test this hypothesis, we here studied transcriptional, metabolic and physiological responses of the TTSs and the WTS in cultivations at 30 °C and 40 °C.

## Results

Three clonal populations of the *S. cerevisiae* strain CEN-PK113-7D were previously evolved through more than 300 generations^18^. Three terminal clones were randomly selected from each population. Seven of these nine strains nearly duplicated the WTS specific growth rate at 40 °C (TTS11, TTS12, TTS13, TTS21, TTS22, TTS31 and TTS33). These strains were send for whole genome sequencing. Since the genome sequence of TTS11, TTS12 and TTS13 were similar, we selected TTS11, TTS21, TTS22, TTS31 and TTS33 for the experiments reported in this study. Physiological, metabolic and transcriptional responses of the TTSs and the WTS to 30 °C and 40 °C were measured under a controlled environment at non-limiting conditions using bioreactors operated at pH 5 and fully aerobic conditions.

### Physiological response to 30 °C and 40 °C

**Figure 1:**
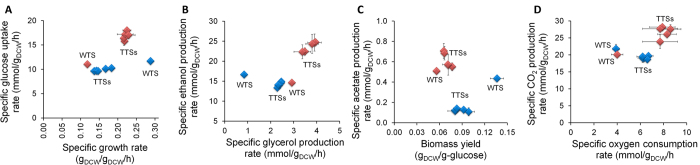
Physiological characterization of wild type and thermotolerant yeasts strains in aerobic cultivations at 30 °C (blue diamonds) and 40 °C (red diamonds) using bioreactors. (**A**) Specific rates of growth and glucose uptake. (**B**) Specific rates of glycerol and ethanol accumulation. (**C**) Specific rates of acetate accumulation and biomass yields. (**D**) Specific rates of oxygen consumption and carbon dioxide production. Averages and standard deviations were calculated from three independent experiments.

Figure 1 shows the quantitative physiological responses of the TTSs and the WTS to 30 °C and 40 °C; the carbon and redox balances are shown in Supplementary Table 1. The different TTSs showed very similar physiological responses to each temperature. The WTS grew nearly three times slower at 40 °C compared with 30 °C, but remarkably, the TTSs showed around 1.4 times higher specific growth rate at 40 °C compared with 30 °C (Fig. 1A). Despite the significant difference between specific growth rate at 30 °C and 40 °C, the WTS consumed glucose at similar rates, whereas the TTSs had an increased glucose uptake rate by of 1.7 times at 40 °C compared to that at 30 °C. The rates of ethanol and glycerol accumulation in the TTSs at 30 °C and the WTS at 40 °C were similar, whereas the WTS growing at 30 °C accumulated glycerol at the slowest rate (Fig. 1B). The TTSs exposed to 40 °C showed the fastest ethanol and glycerol production rates, with ethanol accumulation being around 1.6 times faster than in the TTSs at 30 °C and in the WTS at 30 °C and 40 °C.

Specific acetate accumulation in the WTS at 30 °C was around 4 times faster than in the TTSs at 30 °C, but similar when both strains were grown at 40 °C (Fig. 1C). Heat also provoked lower biomass yields in both set of strains, but this parameter was closer between the TTSs at 30 °C and the WTS at 40 °C. Increased ethanol production in the TTSs at 40 °C showed a positive correlation with the increase in CO_2_ production (Fig. 1D). Remarkably, the TTSs consumed more oxygen than the WTS cultivated at both temperatures (Fig. 1D). These results are consistent with the stoichiometric and reduction balances (Supplementary Table 1).

An exciting insight from these results is the fact that the specific rates of growth, glucose consumption, and ethanol and glycerol production are closer between the TTs and WT strains cultivated at 30 °C and 40 °C, respectively. To get more understanding on the molecular changes underlying these similar physiological responses, we performed global transcriptional analysis of both the TTSs and the WTS at 30 °C and 40 °C.

### Global transcriptional response to 30 °C and 40 °C

**Figure 2:**
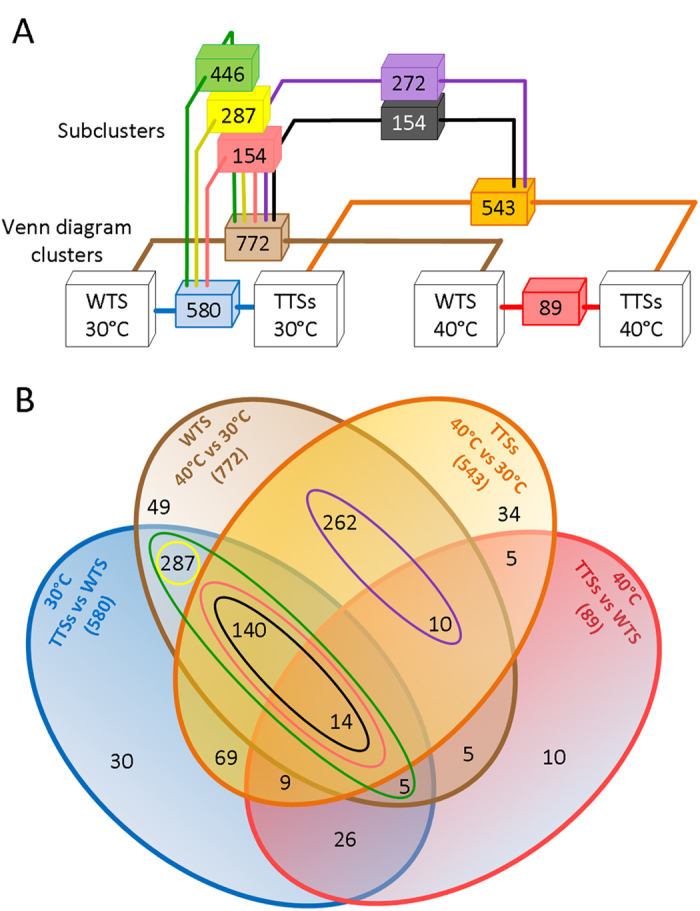
Distribution of global transcriptional changes in the TTSs and the WTS in response to balanced growth at 30 °C and 40 °C. The entire set of genes identified in this analysis (~5708) were clustered based on their significant expression changes (p < 10^−5^) computed from the following contrasts (**A**) TTSs-30 °C vs WTS-30 °C (blue cluster), WTS-40 °C vs WTS-30 °C (brown cluster), TTSs-40 °C vs TTSs-30 °C (orange cluster), and TTSs-40 °C vs WTS-40 °C (red cluster). These clusters were analyzed by a Venn diagram (**B**) and the following relevant junctions/clusters raised up (**A**,**B**): the green cluster of genes with transcriptional changes in both the TTSs-30 °C and WTS-40 °C compared with the WTS-30 °C –heat response genes; the yellow cluster including genes with transcriptional changes exclusively observed in the WTS at 40 °C and the TTSs at 30 °C compared with the WTS at 30 °C –heat response genes fully expressed in the TTSs at 30 °C; the set of 154 genes which changed expression upon exposing of the WTS at 40 °C and the TTSs at 30 °C compared with the WTS at 30 °C (pink cluster), and also upon exposing the TTSs at 40 °C compared with the TTSs at 30 °C (black cluster), this set of heat response genes showed a permanent partial transcriptional change in the TTSs at 30 °C, which was perfected when exposing these strains at 40 °C; and the purple cluster containing genes which expression only changed in both the TTSs and the WTS exposed to 40 °C. It is important to notice that changes in global gene expression of the TTSs were similar (p < 10^−3^), thus the present analysis was performed using the average fold change (Log2).

Microarray transcriptional profiling was used to investigate global changes of gene expression in the five TTSs and in the WTS cultivated at 30 °C and 40 °C. Microarrays were performed on biological triplicates for each condition and strain (36 in total; 6 strains and 2 conditions). The 30 °C, WTS microarrays served as a base to compute relative gene expression changes (fold-change [log2]), in the WTS at 40 °C and in the TTSs at 30 °C (Fig. 2A); the microarrays of the TTSs at 30 °C were used to assess relative gene expression changes in the TTSs at 40 °C; and the microarrays of the WTS at 40 °C served as a base to compute relative gene expression changes in the TTSs at 40 °C. Fold-changes in gene expression among the TTSs growing at the same temperature were similar and thus we used the average fold-change[log2] for the transcriptional analyzes showed here. A Venn diagram of genes with significant expression change is shown in Fig. 2B. Changing the cultivation temperature from 30 °C to 40 °C resulted in expression changes of 772 genes in the WTS, and 543 genes in the TTSs. Most of the genes with transcriptional changes in the WTS at 40 °C, but absent in the TTSs at 40 °C were found to be differentially expressed between the TTSs and WTS at 30 °C (287 genes) –the yellow cluster (Fig. 2A,B). Furthermore, 154 differentially expressed genes in the WTS at 40 °C were observed in the TTSs at 40 °C (black cluster), and also in the TTSs at 30 °C (pink cluster). This suggested that this group of genes changed their expression in the TTSs at 30 °C and also experienced another change when temperature was increased to 40 °C. In sum, 446 genes were differentially expressed in the TTSs at 30 °C and in the WTS at 40 °C compared with the WTS growing at 30 °C (green cluster). There were only 272 genes which transcription was exclusively affected by the high temperature (purple cluster). As a result of all these changes in global transcriptional profiles we observed few changes in gene expression when compared the TTSs and the WTS at 40 °C (89 genes). Therefore, we asked whether the changes in transcriptional profiles observed in the TTSs at 30 °C were part of a core stress response permanently triggered in these strains. Namely, we tested the hypothesis that the direction and magnitude of changes in the expression of these 446 genes found in the TTSs at 30 °C were similar to those observed in the WTS when it was exposed to 40 °C.

### Correlations between transcriptional responses to 30 °C and 40 °C

**Figure 3:**
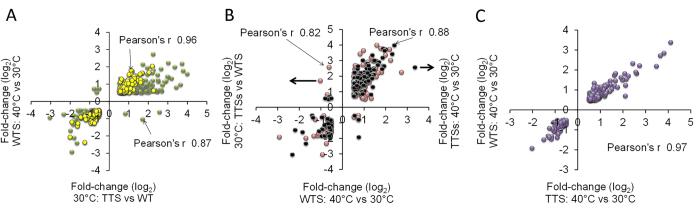
Pearson correlations of global changes in expression of genes grouped in the subclusters show in Fig. 2. (**A**) Changes in expression of heat response genes highly correlate between the WTS at 40 °C and TTSs at 30 °C, compared with the WTS at 30 °C (green and yellow dots respectively) –r = 0.87, P < 0.001 and r = 0.96, P < 0.001. (**B**) The lower correlation observed in the green cluster is due to the fact that a set of 154 genes changed their expression in the TTSs at 40 °C and 30 °C, compared with the WTS at 40 °C (pink and black dots) –r = 0.82, P < 0.001 and r = 0.88, P < 0.001. (**C**) The highest correlation was observed in the group of genes differentially expressed in the TTSs and WTS at 40 °C compared with the TTSs and the WTS at 30 °C, respectively (purple dots) –r = 0.97, P < 0.001.

The global similarity between transcriptional responses in the TTSs and the WTS to 30 °C and 40 °C were evaluated through the Pearson correlation (Fig. 3). As anticipated, the green cluster, comprising 446 differentially expressed genes had good correlation between expression changes in the WTS exposed to 40 °C and in the TTSs exposed to 30 °C (Pearson r = +0.87, P < 0.001). A separate analysis of the 287 differentially expressed genes of the yellow cluster, which is a sub-set of the green cluster (Fig. 2) comprising differentially expressed genes only found in the TTSs at 30 °C and in the WTS at 40 °C, showed an even higher correlation (Pearson r = +0.96, P < 0.001). These results confirmed the existence of a core stress response activated in the TTSs when they were exposed to 30 °C, which is similar to that activated in the WTS when it was grown at 40 °C. Furthermore, it was found that the 154 differentially expressed genes of the pink and black clusters showed good correlation between the TTSs cultivated at 30 °C and 40 °C, respectively compared with the WTS at 40 °C (Pearson r = +0.87, P < 0.001 and +0.82, P < 0.001, respectively), confirming that transcriptional program of this group of genes was operated by phenotypic changes in the TTSs growing at 30 °C (e.g. changes in sterols composition) and by the stress response to heat.

There is also an additional group of genes belonging to the purple cluster that showed similar changes in the TTSs and in the WTS when exposed to 40 °C (Pearson r = +0.97, P < 0.001). Taking together, these results showed that minor changes in the transcriptional program occurred in the TTS at 40 °C since, from the 772 differentially expressed genes in the WTS at 40 °C, 40% had already changed their expression at 30 °C (Fig. 1A), 22% were predominantly changed at 30 °C (Fig. 3B), and 38% changed their expression in response to temperature only (Fig. 3C). We therefore evaluated whether the global average fold change of gene expression changes (Log_2_FC) among specific cellular processes in the TTSs at 30 °C and 40 °C compared with the WTS at 40 °C.

### The core stress response in the TTSs primes for heat stress response to 40 °C

**Figure 4:**
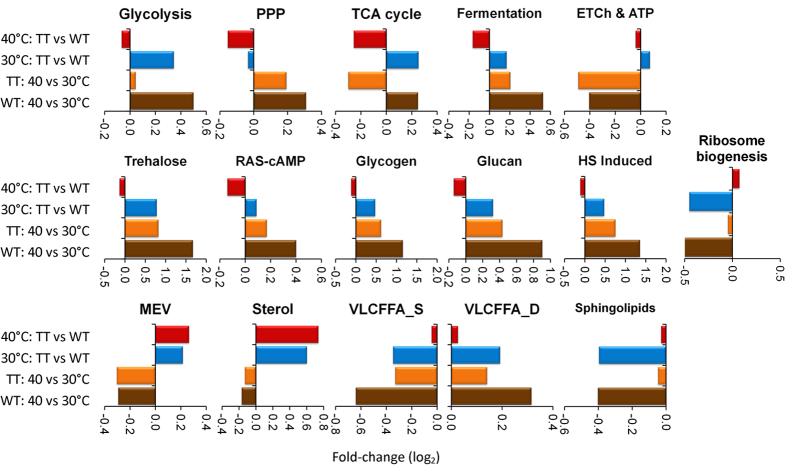
Average relative fold change (Log2) of genes having transcriptional changes in different metabolic pathways and cellular responses to heat shock. The evaluated conditions are those showed in the Venn diagram clusters (Fig. 2).

The transcriptome data were clustered in groups of genes associated to the most representative metabolic and cellular processes reprogramed in response to heat (Fig. 4). The average of the Log_2_ fold change (Log_2_FC) of gene expression in each cluster was calculated for four pair-wise comparisons. Even though a perpetual environmental stress response was activated in the TTSs under optimal temperature (blue bars), a similar global gene expression response can be observed in both the TTSs and the WTS at 40 °C for most of the metabolic and cellular processes (Fig. 4; blue bars + orange bars ≈ brown bars). This is remarkable and have two significances: 1) gene expression program in the yeast at 40 °C have a perfect integral control –namely, the perturbed transcriptional profile of the TTSs at 30 °C reset almost exactly to the ancestral perturbed profile of the WTS at 40 °C; and 2) gene expression program is robust since expression levels of stress related genes to 40 °C work in a very narrow range of perfectly defined transcriptional programs. Thus, global transcriptional changes between the TTSs and the WTS were basically reduced to changes in the expression of genes associated to the mevalonate (MEV) and sterol synthesis pathways (Fig. 4). The down-regulation of the TCA cycle genes in TTSs at 40 °C reflected the diminution of gene expression of mitochondrial TCA genes (Supplementary Fig. 1), which in the WTS were slightly up-regulated at 40 °C. Interestingly, the same profile was observed in the TTSs at 30 °C and in the WTS at 40 °C.

### Functional and transcriptional regulation of differentially expressed genes to 30 °C and 40 °C

Most of the differentially expressed genes of the yellow cluster (Fig. 2) with correspondence between the TTSs at 30 °C and the WTS at 40 °C (Fig. 3A) were down-regulated (171 genes, 60%) (Supplementary Table 2). This group is enriched in genes encoding tRNAs, rRNAs, subunits of the RNA polymerases I and III, ribosomal and processome proteins, amino acids transporters and the *SCH9* gene, among others. *SCH9* encodes a protein kinase required for TORC1-mediated ribosome synthesis, entry into the G0 phase and G1 cyclin (CLN1) activity which promotes the transition from the G1 to S phase of cell cycle. These changes can partially explain the growth trade-off observed in the TTSs at 30 °C (Fig. 1A). Interestingly, the cAMP-dependent protein kinases TPK1 and TPK2 were up-regulated, as well as the alkaline ceramidases YPC1 and YDC1 involved in sphingolipid metabolism and ceramides synthesis. Ceramides participate in cellular signaling to regulate differentiation, proliferation, and programmed cell death^26^.

In the purple cluster comprising the 272 differentially expressed genes with similar expression changes between the TT and WT strains at 40 °C, there are 191 up-regulated genes (70%). Many of them with unknown functions. In this cluster we found genes encoding RAD59 and RAD54 involved in the repair of double-strand breaks in DNA during vegetative growth, HXT5 and HXT7 glucose transporters, HSP30 which is a negative regulator of the H(+)-ATPase PMA1, some catalases and the heat shock protein regulators HCH1 and AHA1. The latter proteins regulate the activity of HSP82 and HSC82 involved in the negative regulation of the heat-sock transcription factor HSF1^27^,^28^.

The 154 genes with differential expression between the WTS at 30 °C and the TTSs exposed to 30 °C and 40 °C (pink and black clusters in the Figs 2 and 3B), included 108 up-regulated genes (70%). This group is enriched in genes encoding proteins required for trehalose metabolism, and glycogen and glucan synthesis, as well as heat shock proteins. The *ROM1* gene encoding a GDP/GTP exchange protein for RHO1 which regulates glucan synthesis, and the transcriptional repressor NRG2 that mediates glucose repression and negatively regulates filamentous growth were also upregulated, as well as HES1 that regulates sterol biosynthesis. 76% (82 genes) of these 108 up-regulated genes are under the control of the transcription factors HSF1 (heat-shock stress), MSN2/MSN4 (general stress), YAP6 (osmotic stress), YAP1 (oxidative stress) and the transcriptional activators SWI4-SWI6 (cell wall stress). These TFs also regulate 40% (107 genes) and 33% (95 genes) of the genes with transcriptional reprograming in the TTSs and the WTS at 40 °C (purple cluster), and in the TTSs and the WTS at 30 °C and 40 °C, respectively (yellow cluster). Most of the genes controlled by HSF1 are also cross-regulated by MSN2/4 and YAP6.

## Discussion

### Global metabolic adjustments to 30 °C and 40 °C

These results suggested that metabolism of all the strains was a mix of fermentation and respiration (Fig. 1 and Supplementary Table 1), since differences between total CO_2_ and ethanol accumulation is positive. Remarkably, respiration and glucose consumption rates were similar between the WTS at both temperatures, whereas glycerol accumulation rate increased in the WTS at 40 °C. This suggest that under high temperature, the WTS did not produce energy by respiratory metabolism, despite the fact that O_2_ consumption was similar to 30 °C. Similar alterations can be observed when comparing the WTS at 30 °C and the TTSs at 30 °C and 40 °C. However, the TTSs duplicated the O_2_ uptake rate at 40 °C. According to these results, it has been reported that the yeast strain CEN-PK113-7D cannot generate ATP above 37 °C^6^, although oxygen consumption is higher at 38 °C compared with 30 °C^7^.

The contribution of glycerol as a sink for NADH excess produced during glycolysis is not enough to close the redox balance (Supplementary Table 1, and Supplementary Fig. 1). Thus, the excessed redox power could be released through the oxidation of cytosolic NADH by the external dehydrogenases NDE1 and/or NDE2^29^. The latter was up-regulated in the TTSs at 30 °C and in the WTS at 40 °C. Indeed, the external dehydrogenases and the glycerol shunt through GUT2 are necessary and complementary for oxidation of cytosolic NADH^30^. However, under impaired ATP synthesis, the electron transport chain may finalize with the donation of electrons to the O_2_. In the TTSs and the WTS cultivated at 40 °C, most of the ATPase biogenesis genes were down-regulated (Supplementary Fig. 1). Interestingly, some genes encoding proteins required for heme A synthesis, an essential co-factor of cytochrome C oxidase, were up-regulated in the TTSs at 30 °C and the WTS at 40 °C; including COX10 which transfers farnesyl, the final product of the MEV pathway, into protoheme to synthesize the heme A, and COX17, a copper metallochaperone which delivers copper into the cytochrome C oxidase^31^. Furthermore, the high-affinity copper transporters CTR1 and CTR3 were up-regulated in the TTSs at 30 °C and the WTS at 40 °C. It was also observed that PFK27 and PFK26, which catalyze the synthesis of fructose-2,6-bisphosphate (F2,6bP) from 6-phosphofructo-2-kinase^32^, were both up-regulated at 40 °C in the TTs and WT strains. F2,6bP is a strong positive allosteric effector of the phosphofructokinase (PFK1 and PFK2), and regulates glycolysis accordingly^33^.

### Global transcriptional adjustments to 30 °C and 40 °C

**Figure 5:**
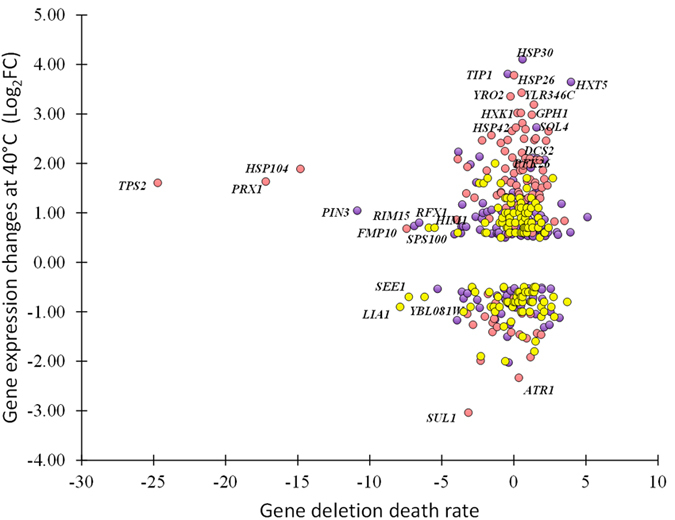
Correlations between sensitivity to heat shock and fold change expression of genes grouped in red, blue and purple clusters shown in the Fig. 2. We use data on the deletion strains heat sensitivity from Gibney *et al.*^34^, who calculated the death rate for experiments where cultivation of single deletion strains at 30 °C were exposed to 50 °C.

Some genes with differential expression upon temperature increase appear to be only needed for acquiring thermotolerance whereas others are required for innate thermotolerance^5^,^34^. Many of them are not essential for heat survival^34^. Here we showed that 772 and 543 genes changed their transcriptional profile when the WTS and the TTSs were cultivated at 40 °C (Fig. 2), but 287 genes kept changed expression at 30 °C in the TTSs with the similar transcriptional program observed in the WTS at 40 °C (Fig. 3A). Furthermore 154 genes showed correlated differential expression between the TTSs at 30 °C and 40 °C and the WTS at 30 °C and 40 °C (Fig. 3B). Interrogating our results with the results on gene deletion death rate^34^, we found that 53% of the genes with differential expression in the TTSs and the WTS are required for thermotolerance (Fig. 5 and Supplementary Table 3). Among these genes, the trehalose phosphate synthase *TPS2*, the heat shock protein disaggregase *HSP104*, and the mitochondrial peroxiredoxin *PRX1* which is induced under oxidative stress. Indeed, 60% of the differentially regulated genes on the pink and purple clusters have significant contribution to heat tolerance (Fig. 5). Many genes with transcriptional changes upon temperature up-shift in the WTS and in the TTSs at 30 °C are apparently less required for thermotolerance (yellow cluster). The small heat shock proteins HSP26 and HSP42 and the HSP30, were up-regulated but seems not to be required for thermotolerance^34^.

### Tuning gene expression changes for rapid adaptation to heat

Expression programs for stress related genes are characterized by distinct transcriptional mechanisms and high levels of noise in their expression patters^2^. Here we found that genes with high transcriptional noise were stress related genes enriching the pink, black and purple clusters (Figs 2 and 5), mainly governed by the stress transcription factors HSF1, MSN2/MSN4, YAP6 and YAP1 (Supplementary Table 2). Whereas genes related to biosynthetic processes found in the red cluster showed less noise (Fig. 5).

In cells exposed to different simultaneous stresses, the total cellular responses approximate the sum of each individual stress, suggesting that gene expression programs can be combined for precise response in a narrow limit^2^,^5^,^35^. Besides contributing as a preventive heat stress response, the core stress response genes in the green cluster seems to offer a cross protection against high osmolarity and elevated ethanol concentrations^15^. Interestingly, this core may not protect against reactive oxygen species. This protection may come from the purple cluster which transcription changed at 40 °C in all the strains (Supplementary Table 2), since the response to heat offers a cross protection against oxidative stress^36^.

In analogy to the anticipated protection that a mild stress stimulates to prevent detrimental effects of an onslaught version of heat stress^4^,^36^, the core stress response activated by the TTSs at optimal ancestral temperature (30 °C) also appears to be associated to their rapid adaptation to 40 °C (Supplementary Fig. 2). Indeed, to get stationary growth at 40 °C (namely, an unchanged specific growth rate in time), the TTSs required around 8 generations (two sub-cultivations) compared with the 20 generations (eight sub-cultivations) needed by the WTS. If the trajectory of the growth rate reflects the trajectory of homeostatic responses, the TTSs have a short path between global responses at 30 °C and the required homeostatic responses to 40 °C, and probably to high osmolality and ethanol concentrations^15^.

Taken together, these results provide a systems level elucidation of mechanisms for adjusting gene expression and metabolic programs in response to heat through evolutionary timescales. Rapid and efficient adaptation of the TTSs to heat is finely tuned through a permanent activation of a core stress response at 30 °C and a perfected stress response when exposing to 40 °C, partially explaining the observed growth trade-off at 30 °C and the preadaptation to other stresses^15^. Remarkably, these changes appeared after a small change in the chemistry of its major sterol^18^.

## Materials and Methods

### Strains

The thermotolerant yeast strains were previously isolated from ALE experiments carried out by serial dilution of three independent clonal populations of the parental yeast strain CEN-PK113-7D cultivated at 39.5 ± 0.3 °C in minimal media^18^. This procedure was repeated until a significant change in growth rate was detected after more than 300 generations, when three strains were randomly selected from every population. From these nine strains (TT11, TT12, TT13, TT21, TT22, TT23, TT31, TT32 and TT33), seven were sent for genome sequencing. The genome sequence of strains TTS11, TTS12 and TTS13 were similar, and we therefore selected TTS11, TTS21, TTS22, TTS31 and TTS33 for the experiments reported in this study.

### Culture media

The thermotolerant yeast strains and the parental strain were cultivated in shake flasks and bioreactors using minimal media with glucose. This contained 2% glucose and 5 g (NH3)2SO4, 3 g (NH4)2PO4 and 0.5 g 21 MgSO4 per liter, in addition to 1 mL of trace elements solution and 1 mL of vitamin solution. The trace element solution contained, per liter (pH = 4): EDTA (sodium salt), 23 15.0 g; ZnSO4-7H2O, 4.5 g; MnCl2-2H2O, 0.84 g; CoCl2-6H2O, 0.3 g; CuSO4-5H2O, 0.3 24 g; Na2MoO4-2H2O, 0.4 g; CaCl2-2H2O, 4.5 g; FeSO4-7H2O, 3.0 g; H3BO3, 1.0 g; and KI, 25 0.10 g.). The vitamin solution contained, per liter (pH = 6.5): biotin, 0.05 g; p-amino 26 benzoic acid, 0.2 g; nicotinic acid, 1 g; Ca-pantothenate, 1 g; pyridoxine-HCl, 1 g; 27 thiamine-HCl, 1 g and myo-inositol, 25 g. Initial pH of the medium was adjusted to 5.2.

### Cultivation in bioreactors

Cultivations of each of the five evolved strains and the parental strain were performed in triplicates under fully aerobic conditions in 1.0 L vessels using the Dasbox System (DASGIP, Jülich, Germany), containing 0.7 L of minimal media. Temperature was controlled to 30 °C or 40 °C, agitation was kept constant at 500 revolutions per minute and dissolved oxygen (DO) tension was kept at concentrations higher than 40% of air saturation. Automatic control of pH at 5.0 was carried out with the addition of 2 M KOH solution. Prior to cultivation in bioreactors, the strains were grown at the target temperature for more than 20 generations to avoid the effects of innate heat-shock response and population dynamics of yeast adaptation to the temperature.

### Analytics

Cultivation samples were taken from bioreactors and processed at 4 °C every 1–2 hrs. Cells growth was indirectly followed by the sample optical density determined at 600nm. To analyze growth based on cell dry weight (CDW), samples were vacuum filtered and the biomass was washed twice with isotonic solution, dried in a microwave oven for 15 min at medium power and kept in a desiccator until constant weight. Supernatant was used to quantify fermentation metabolites by HPLC, using an Aminex HPX-87H column (Bio-Rad, California, USA) connected to refractive index and photodiode array detectors. The mobile phase consisted of an 8 mM H_2_SO_4_ solution fluxed at 0.5 mL/min, and the assay was run at 50 °C. Pure glucose, organic acids, glycerol and ethanol from Sigma Aldrich were used to construct the calibration curves used to quantify these compounds in the culture.

### Total RNA extraction, analysis

10 mL Samples were taken in the middle of exponential phase of cultivation in bioreactors at 30 °C and 40 °C. These were rapidly put on ice and centrifuged at 3000 g for 5 min and 4 °C. Supernatant was rapidly discarded and biomass pellet was frozen in liquid nitrogen, and then stored at −80 °C. Total RNA was extracted from pellets using the RiboPure™-Yeast Kit (Ambion, TX, USA) with the help of RNAse free solvents (Ambion, TX, USA). Affymetrix Yeast Genome 2.0 Array was used for transcriptome analysis.

In total, we processed 36 arrays from independent triplicates of the five TTSs and the WTS cultivated at either 30 °C and 40 °C. The CEL-files of array data were preprocessed with the Bioconductor^37^ using R software version x64 2.11.1. The Affymetrix chip description file (CDF-file) was obtained from the microarray developers and imported to R using the Bioconductor package makecdfenv. The raw data was normalized using Probe Logarithmic Intensity Error (PLIER) normalization^38^ using 6 only perfect match probes (pm-only). Correction for multiple testing of p-values was done using the Benjamini-Hochberg’s method^39^. The cut off <0.05 was used for adjusted p-value to get differentially expressed genes between each of the four conditions (WTS at 30 °C and 40 °C, and TTSs at 30 °C and 40 °C). The PIANO package was used for reporter GO-term and gene set enrichment analysis^40^.

### Metabolic flux analysis

We used the genome scale metabolic model (GEM) iIN800 to perform metabolic flux analysis. The RAVEN Toolbox^41^ and the Random Sampling algorithms^42^ were used to identify enzymes which showed significant changes in their expressions at 30 °C and 40 °C in both the wild type and TTSs and the rate of their associated reactions. The GEM was first constrained with external fluxes obtained from cultivations in bioreactors (e.g. specific rates of growth, glucose consumption and ethanol production). We did not use specific rates of CO_2_ production and O_2_ consumption to constrain the GEM. The constrained GEM was transformed into a SBML model which was used in simulations with the Random Sampling toolbox to calculate a two columns matrix with the average of fluxes and variances for every reaction. These results were used to calculate standard deviations between two conditions and get Z scores for variations in every reaction of the GEM. Scores from the Student t analysis of gene expression between every experimental condition were used to calculate p-values. Both Z scores and p-values were used to identify reactions showing better correlation between the flux and the expression changes in wild type and TTS.

## Additional Information

**How to cite this article**: Caspeta, L. *et al.* Thermotolerant yeasts selected by adaptive evolution express heat stress response at 30 °C. *Sci. Rep.* **6**, 27003; doi: 10.1038/srep27003 (2016).

## Supplementary Material

Supplementary Information

Supplementary Dataset 1

Supplementary Dataset 2

Supplementary Dataset 3
