# Supplementary material for: Thermotolerant yeasts selected by adaptive evolution express heat stress response at 30 °C
Source: Sci Rep. 2016 May 27;6:27003. doi: 10.1038/srep27003 (PMC4882594; doi:10.1038/srep27003)
Supplement: Supplementary Information [file srep27003-s1.pdf]

Thermotolerant yeasts selected by adaptive evolution express heat stress response at 30°C

Luis Caspeta, Yun Chen and Jens Nielsen*

Supplementary Figures

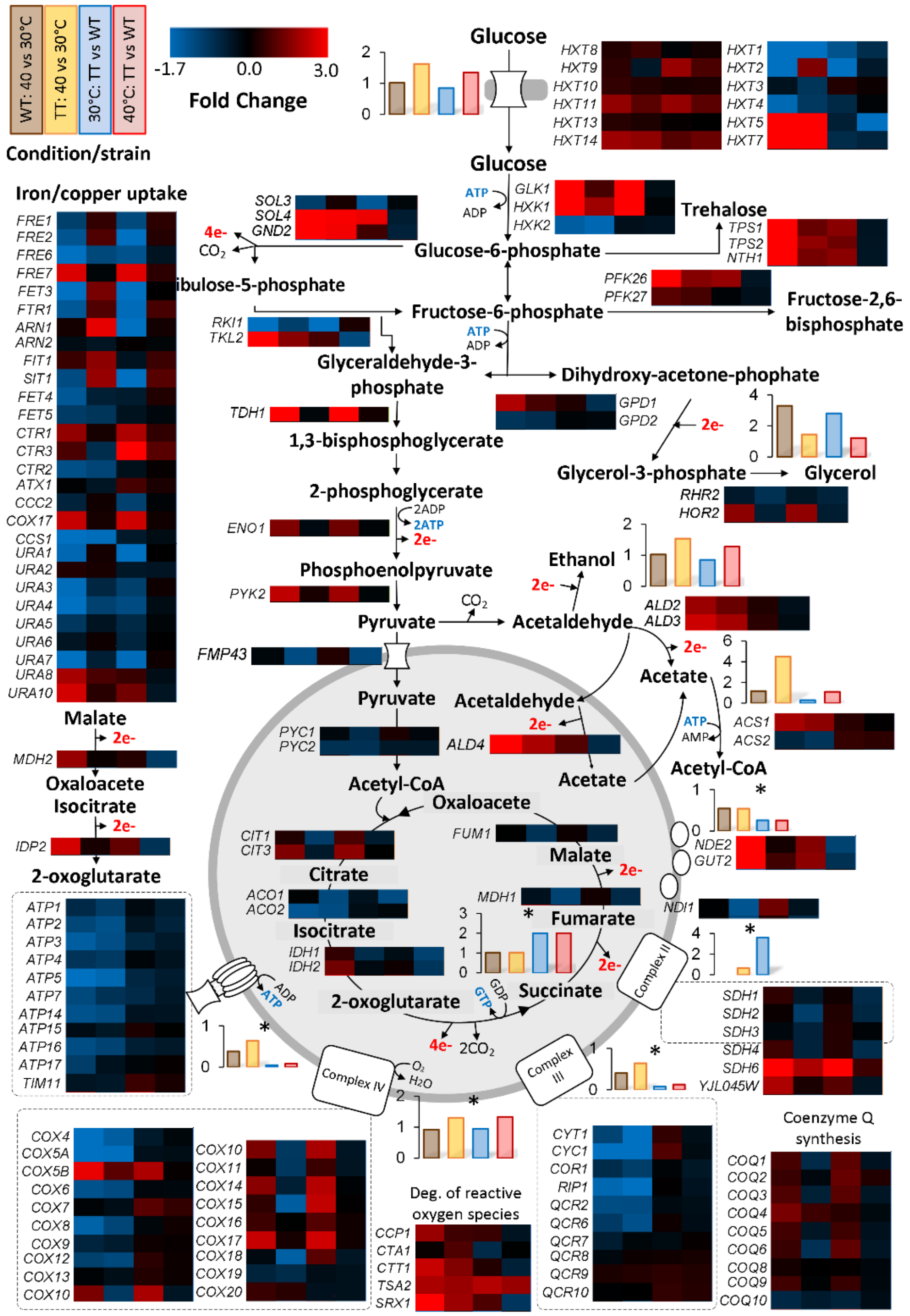

Supplementary Fig. 1 Changes in gene expression and metabolic fluxes in the TT and WT strains cultivated at 30°C and 40°C. Flux balances shown in the bar charts were calculated from data shown in the Fig. 1 or predicted by genome-scale metabolic flux analysis using experimental fluxes as constrains (charts with an asterisk).

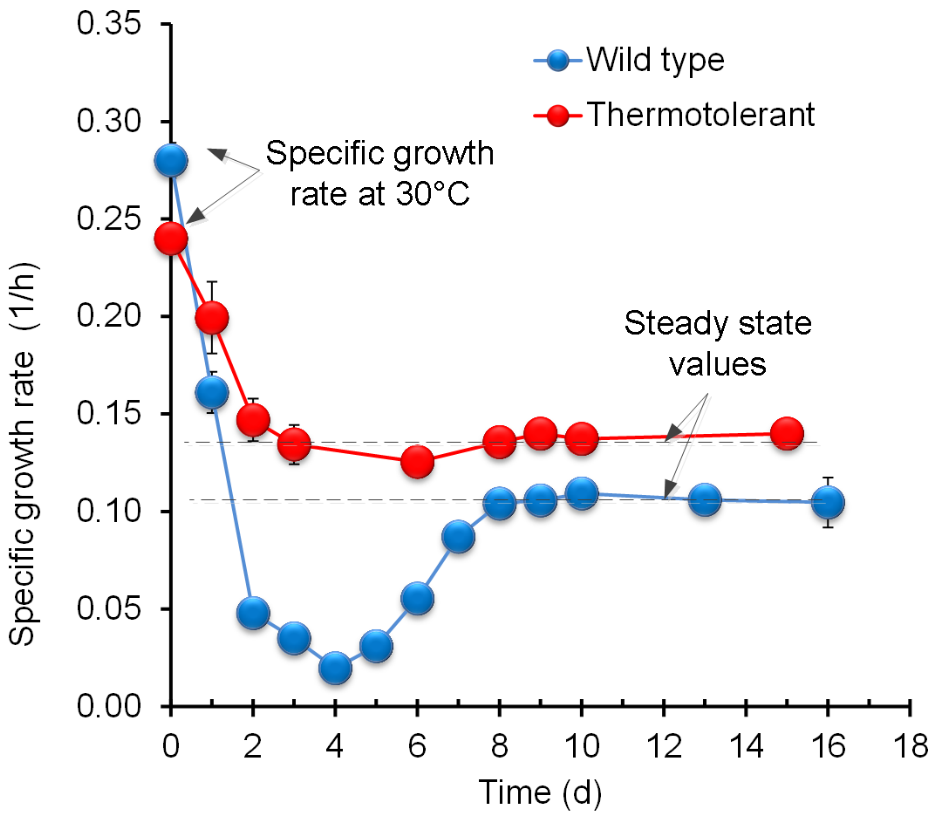

Supplementary Fig. 2 Specific growth rate kinetics of the TTSs and the WTS cultivated at 40°C. Yeast cells from both the TT and the WT strains were first cultivated at 30°C for around 20 generations, showing the specific growth rate pointed up by the arrows. Then, cultivations were transferred to 40°C and daily sub-cultivated in fresh media during 15 to 16 days. The specific growth rate was daily measured in both the WTS and the TTSs. The steady state of the specific growth rate, namely the unchanging specific growth rate in time, was first accomplished by the TTSs after 3 days (~8 generations), while the WTS reached this after 8 days (~20 generations).
